# Applying the Bradford Hill criteria in the 21st century: how data integration has changed causal inference in molecular epidemiology

**DOI:** 10.1186/s12982-015-0037-4

**Published:** 2015-09-30

**Authors:** Kristen M. Fedak, Autumn Bernal, Zachary A. Capshaw, Sherilyn Gross

**Affiliations:** Department of Environmental and Radiological Health Sciences, Colorado State University, 350 West Lake Street, Fort Collins, CO 80521 USA; Cardno ChemRisk, 130 Vantis Suite 170, Aliso Viejo, CA 92656 USA; Cardno ChemRisk, 4840 Pearl East Circle, Suite 300 West, Boulder, CO 80301 USA

**Keywords:** Causation, Causal inference, Data integration, Bradford Hill, Molecular epidemiology

## Abstract

In 1965, Sir Austin Bradford Hill published nine “viewpoints” to help determine if observed epidemiologic associations are causal. Since then, the “Bradford Hill Criteria” have become the most frequently cited framework for causal inference in epidemiologic studies. However, when Hill published his causal guidelines—just 12 years after the double-helix model for DNA was first suggested and 25 years before the Human Genome Project began—disease causation was understood on a more elementary level than it is today. Advancements in genetics, molecular biology, toxicology, exposure science, and statistics have increased our analytical capabilities for exploring potential cause-and-effect relationships, and have resulted in a greater understanding of the complexity behind human disease onset and progression. These additional tools for causal inference necessitate a re-evaluation of how each Bradford Hill criterion should be interpreted when considering a variety of data types beyond classic epidemiology studies. Herein, we explore the implications of data integration on the interpretation and application of the criteria. Using examples of recently discovered exposure–response associations in human disease, we discuss novel ways by which researchers can apply and interpret the Bradford Hill criteria when considering data gathered using modern molecular techniques, such as epigenetics, biomarkers, mechanistic toxicology, and genotoxicology.

## Background

In 1965, Sir Austin Bradford Hill gave the first President’s Address to the newly formed Section on Occupational Medicine, which was published within the Proceedings of the Royal Society of Medicine [1]. Hill began his address by pointing out a fundamental problem facing the Section members: how could they effectively practice preventative occupational medicine without a basis for determining which occupational hazards ultimately cause sickness and injury? Namely, Hill asked, “In what circumstances can [one] pass from [an] observed *association* to a verdict of *causation*?” [1]. He proceeded to propose nine “aspects of association” for evaluating traditional epidemiologic data. These aspects, which have since become fundamental tenets of causal inference in epidemiology, are often referred to as the Bradford Hill Criteria.

The nine “aspects of association” that Hill discussed in his address (strength of association, consistency, specificity, temporality, biological gradient, plausibility, coherence, experiment, and analogy) have been used to evaluate countless hypothesized relationships between occupational and environmental exposures and disease outcomes. Yet, when Hill conceived these nine aspects (hereafter referred to as criteria), the mechanistic connections between exposure and disease were not well understood. Consider that Hill published his criteria just 12 years after Watson and Crick first suggested the double-helix model for DNA. Traditional epidemiologic study designs that were developed and used around the time of Hill’s speech treated the connection between exposure and disease as a ‘black box’—meaning that the biological mechanisms that occur between exposure and disease onset were unknown and therefore omitted in study design [2]. Over the past 50 years, advances in scientific fields (e.g., molecular genetics, genomics, molecular toxicology) and technology (e.g., computers, software, statistics, analytical methods) have provided researchers with a much deeper and more complex understanding of how diseases initiate and progress, effectively allowing researchers to glimpse into the ‘black box’ of the exposure-to-disease paradigm. As a result, researchers considering causal inference have new and more diverse types of information to consider when establishing causality beyond the traditional epidemiologic study designs that were available when Hill wrote his causal criteria.

Data integration refers to the incorporation of data, knowledge, or reasoning from across multiple disciplines or approaches, with the goal of generating a level of understanding or knowledge that no discipline achieved alone [3, 4]. Data integration, while not always referred to by that term, has been discussed in light of causal inference of disease for over a decade, and the epidemiologic community has generally welcomed these interdisciplinary collaborations [5–7]. For example, the preface of the 5th edition of the *Dictionary of Epidemiology* directly acknowledges the “positive blurring of the boundaries of epidemiological research methods” into other scientific disciplines. The preface welcomes non-epidemiologists to contribute to and use the *Dictionary* and inversely invites trained epidemiologists to utilize the concepts within the *Dictionary* in non-epidemiological initiatives [4]. Furthermore, numerous agencies, organizations, and academics have recently attempted to establish frameworks or guidelines for data integration in the field of human health and ecological risk assessment. These frameworks consider how researchers should address, compare, and contrast the value and contributions of data that come from different evidence streams or scientific disciplines [8–11].

Hill aptly stated at the end of his speech that “[a]ll scientific work is incomplete… [and] liable to be upset or modified by advancing knowledge” [1]. Today, researchers considering causal inference must integrate data from a variety of scientific disciplines. Herein, we discuss how data integration in the field of causal inference of diseases affects the application and interpretation of each of Hill’s criteria.

## Criteria 1: strength of association

Hill’s first criterion for causation is *strength of the association*. As he explained, the larger an association between exposure and disease, the more likely it is to be causal. To illustrate this point, Hill provided the classic example of Percival Pott’s examination of scrotal cancer incidence in chimney sweeps. The tremendous strength of association between that occupation and disease—nearly 200 times greater than seen in other occupations—led to a determination that the chimney soot was likely a causal factor. Contrarily, Hill suggested that small associations could more conceivably be attributed to other underlying contributors (i.e. bias or confounding) and, therefore, are less indicative of causation.

Defining what constitutes a “strong” association is critical to the assessment of potentially causal relationships. Advances in statistical theory and the computational processing power have allowed scientists to delineate strong versus weak associations using more defensible mathematical criteria than Hill had in mind. Strength is no longer interpreted as simply the magnitude of an association. Furthermore, researchers have gained a greater appreciation for multi-factoral diseases and the existence of determinant risk factors that are small in magnitude yet statistically strong. Today, statistical significance—not the magnitude of association—is the accepted benchmark for judging the strength of an observed association, and thus its potential causality.

Yet, these same statistical and computational advances necessitate an added degree of scrutiny when interpreting study results. Modern tools have enabled researchers to collect much larger datasets, access wide ranges of metadata, employ complex algorithms, and choose from a multitude of statistical approaches. As such, statistically significant results presented within a study are not always biologically meaningful or methodologically appropriate for contributing to causal inference. Conversely, failure to mathematically demonstrate statistical significance in a single study does not preclude the possibility of a meaningful exposure–response relationship in reality. Thus, assessing *strength of association* in causal inference requires examination of underlying methods, comparison to the weight of evidence in the literature, and consideration of other contextual factors including the other criteria discussed herein.

An example can be seen in the analysis and subsequent re-analysis of pulmonary function in a cohort of 106 workers at a flavorings manufacturing facility that used a variety of chemicals, including acetaldehyde, acetoin, benzaldehyde, butyric acid, and diacetyl [12, 13]. In the original study conducted by the National Institute for Occupational Safety and Health (NIOSH), researchers retrospectively analyzed spirometry reports and job title records collected by the cohort’s employer [13]. The authors presented statistically significant effect estimates showing that employees in jobs with higher potential for flavoring chemical exposures had 2.8 times greater annual declines in forced expiratory volume (FEV) than employees in lower-exposure jobs. This led authors to conclude that there was a statistically strong association between occupational exposure to flavorings and restrictive pulmonary disease [13]. However, as Ronk et al. [12] pointed out, the NIOSH researchers did not account for the inherently correlated nature of the longitudinal spirometry test data in their choice of regression analysis, which would affect the data variability and therefore standard error estimates and subsequent statistical inference [14, 15]. Ronk and colleagues re-analyzed the same data set using generalized estimating equations (GEE) that account for these correlations and did not find any statistically significant associations [12]. The varied outcomes and author interpretations associated with these two studies underscores how the use of different statistical methods can lead to statistically different results, thus impacting the application of *strength of association*.

## Criteria 2: consistency

Traditionally, Hill’s *consistency* criterion is upheld when multiple epidemiologic studies using a variety of locations, populations, and methods show a consistent association between two variables with respect to the null hypothesis. Hill stressed the importance of repetitive findings because a single study, no matter how statistically sound, cannot be relied upon to prove causation due to ever-present threats to internal validity. This criterion is still very appropriate for determining causal relationships; however, data integration practices have led to an evolution in thought on what constitutes consistency. The concept of data integration is inherently influential in the interpretation of the *consistency* criterion as it speaks to understanding a consistent story across multiple disciplines or practices. For example, through the lens of data integration, molecular experimentation can bolster epidemiologic findings by providing supportive evidence for a mechanistic hypothesis, thereby lessening the need for repetition among numerous observational studies. In vitro toxicology studies that suggest a mode of action such as genotoxicity or altered gene expression can support an association found in an epidemiologic study. By integrating results from multiple types of studies, researchers can show consistency in the causal story by illuminating various mechanistic points along the exposure-to-effects paradigm. This is a much broader interpretation of *consistency* than Hill’s original concept of repetitive epidemiologic findings.

The story of benzene-associated Acute Myeloid Leukemia (AML) illustrates the application of the *consistency* criterion in light of modern data integration. Both animal models and in vitro human cell cultures demonstrated that hydroquinone and para-benzoquinone are the active metabolites of benzene [15, 16]. Additionally, it was shown that hydroquinone induces cell changes that are consistent with various cellular changes known to mark the early progression of AML in humans [16, 17]. These molecular-level studies supported available human in vivo data (i.e., standard epidemiological studies), thereby lessening the need for additional observational studies to support a causal relationship.

Similarly, data integration played a role in the demonstration of consistency to support a causal relationship between polychlorinated biphenyl (PCB) exposure and melanoma. Consistency among epidemiologic studies of PCB exposure and melanoma, and in vitro mechanistic studies with human melanocytes support a plausible mechanism by which PCBs disrupt melanogenesis [18, 19]. Collectively, these data contributed to the decision by the International Agency for Research on Cancer Monograph Working Group to upgrade PCBs to a Group 1 carcinogen [18, 20]. Consistency between rodent and human bioassays also demonstrates support for a mechanism of carcinogenicity via initial binding to the aryl-hydrocarbon receptor (AhR) by PCB 126 and 2,3,7,8-tetrachlorodibenzo-para-dioxin, (TCDD) in other cancers [18, 20]. These examples illustrate how advanced molecular analyses can be integrated with the results of observational studies to demonstrate consistent research findings supporting a potentially causal relationship.

## Criteria 3: specificity

Hill suggested that associations are more likely to be causal when they are specific, meaning the exposure causes only one disease. While Hill understood that some diseases had multiple causes or risk factors, he suggested that “if we knew all the answers we might get back to a single factor” responsible for causation. This view is indicative of the fact that, in Hill’s era, exposure was often defined in terms of proxies for true exposures, such as an occupational setting or a residential location. Today, we attempt to specifically define exposures not in terms of a person’s surroundings or conditions, but rather as an actual dose of a chemical, physical, or biological agent. While some examples of highly specific agent-outcome associations exist, most exposure and health concerns at the forefront of research today center around complex chemical mixtures and low-dose environmental and occupational exposures complicated by a variety of risk factors.

The original criterion of *specificity* is widely considered weak or irrelevant from an epidemiologic standpoint. However, *specificity* may have new and interesting implications in the broader context of data integration. For example, researchers can demonstrate a molecular mechanism of action with precisely defined (i.e., specific) relationships between the agent and the effects using a variety of research methodologies. Asbestos exposure and the development of asbestosis is one example. In addition to the common use of occupational history as a surrogate for asbestos exposure in an epidemiological framework, advances such as refined standardized criteria for clinical diagnosis of asbestosis, microscopic lung fiber burden analyses and identification of asbestos bodies, as well as increased understanding of the relative potency of different fiber types have further clarified how asbestosis may be specifically caused by asbestos exposures [21–24]. With data integration, *specificity* evolves into a more powerful criterion, and the lack of specificity can help to narrow down specific agents associated with disease. For example, complex mixtures of chemicals (e.g., tobacco smoke) typically lack specificity when studied using classic epidemiology designs, since multiple diseases can result from the exposures. However, it is possible that data integration may elucidate some mechanistic specificity among the varied disease endpoints associated with these complex carcinogenic mixtures.

## Criteria 4: temporality

*Temporality* is perhaps the only criterion which epidemiologists universally agree is essential to causal inference. Consider that Rothman and Greenland, despite finding a lack of utility or practicality in any of the other criteria, referred to *temporality* as “inarguable” [25]. Hill explained that for an exposure-disease relationship to be causal, exposure must precede the onset of disease. Thus, epidemiologic study designs which ensure a temporal progression between the two measures are more persuasive in causal inference.

When ensuring temporality in the context of modern-day environmental exposures, it is important to consider that many of these involve low levels of exposure over extended time frames, and low incidence, micro-scale outcomes that occur following long latency periods. These factors make the prospect of designing a traditional epidemiologic study in which temporality is firmly established a costly, time consuming, and potentially unfeasible task. However, improved chemical exposure monitoring and analytical capabilities, molecular epidemiology techniques, and advances in understanding disease progression allow for new and expanded ways to meet this criterion across a variety of study designs. The use of biomarkers, state-of-the-art analytical testing at low limits of detection, and understanding of windows of toxicity and chromosome abnormalities in disease progression have increased our confidence in *temporality* as a useful criterion.

A modern example of expanded temporal analysis using data integration is illustrated by studies of low-dose exposures to arsenic through drinking water and food. Arsenic levels in hair and nails serves as a biomarker of past exposure [26, 27], and drinking water analytical records from an individual’s past and present residences can be used to create an estimate of historic environmental exposure [28]. Limited windows of exposure can be evaluated to determine effects of exposure during sensitive stages [29, 30]. By integrating new data and knowledge from these tools, temporal relationships can be considered even within cross-sectional or ecological studies that do not implicitly establish temporality within the study design.

Today, our understanding of temporality now includes a wider range of precisely defined wider exposure windows, some of which are more relevant to disease outcomes than previously thought. Through epigenetic mechanisms (i.e., DNA methylation, histone modifications), exposures that occur during specific periods of development or even in previous generations can result in phenotypic differences in offspring [31]. Such changes could be responsible for generational effects of synthetic estrogen diethylstilbestrol (DES) exposure which can lead to increased risk of breast cancer multiple generations removed from the initial exposure [32]. Analytical techniques are improving to detect these changes and to determine which epigenetic alterations may serve as indicators of disease potential and persistent biomarkers of a previous exposure [33]. Understanding the molecular-level changes that precede an observable outcome can help establish the temporal progression in a multigenerational causal story [34].

## Criteria 5: biological gradient

Hill wrote that “if a dose response is seen, it is more likely that the association is causal.” According to the traditional interpretation of *biological gradient*, the presence of a dose–response relationship supports the causal association between an exposure and an effect [25, 35]. In traditional epidemiology, a monotonic biological gradient, wherein increased exposure resulted in increased incidence of disease, provides the clearest evidence of a causal relationship. However, Hill acknowledged that more complex dose–response relationships may exist, and modern studies have confirmed that a monotonic dose–response curve is an overly simplistic representation of most causal relationships. In fact, most dose–response curves are non-linear and can even vary in shape from one study to the next depending on unique characteristics of the given population, exposure routes, and molecular endpoints assessed [36]. Furthermore, individual susceptibility and synergistic or antagonistic effects of cumulative exposures can make some biological gradients even more difficult to characterize. An example of this effect can be seen in aryl hydrocarbon receptor (AhR)-based mechanisms: many exogenous and endogenous agents can act as partial agonists/antagonists of AhR, and thus modulate the dose–response effect of 2,3,7,8-tetrachlorodibenzodioxin (TCDD) which affects gene expression via AhR [9]. Integration of advanced statistical capabilities, data modeling techniques, and knowledge from increased understanding of biomolecular interactions have resulted in the descriptions of more defined dose–response curves, capable of showing molecular effects at very low levels of exposure. Additionally, growing knowledge of genetic polymorphisms has illuminated the reasons behind individual variations in biological response to toxic insult and the dose–response relationships [8].

It is now possible to observe threshold responses in the low-dose range, rather than assuming linearity for all substances. Furthermore, experimental support for a dose–response phenomenon referred to as hormesis has increased with improved molecular techniques. Hormesis is characterized by low dose stimulation and a high dose inhibition [37]. The dose–response curve associated with this phenomenon is biphasic and, depending on the endpoint measured, is either J or U shaped [38]. Hormesis has been observed in both toxicology and pharmacology, and the features of the observed dose–response are consistent and independent of the biological model, endpoint measured, chemical or physical stressor, and mechanism [37]. The most distinctive feature of hormesis is that it is repeatedly observed below the typical threshold dose [37].

*Biological gradient* is an example of how data integration can complicate causal inference. New tools and technical capabilities have allowed researchers to characterize a variety of low-level molecular endpoints that may not lead to disease or observable adverse outcomes on a larger scale. For example, innate responses can repair, eliminate, or reverse molecular changes caused by low levels of exposure. Thus, molecular changes within the no-observable-adverse-effect level (NOAEL) may not contribute to disease and are more indicative of a threshold dose response. Understanding the mechanisms at low level exposures allows us to elucidate a dose–response curve. For example, the in vitro endpoints for asbestos toxicity include generation of oxidative stress which results in genotoxicity and chromosome damage via DNA adduct formation [39]. However, damage at low levels, while measurable in vitro, is removed via cellular apoptosis which represents adaptive response and a threshold effect. Thus, responses at these low levels may not be indicative of disease, but rather adaptive responses that indicate a threshold must be overcome prior to disease initiation.

Additionally, modern analytics have shown that epigenetic endpoints can occur in the low-dose range of environmental chemical exposures, though these measured changes may not lead to observable disease. For example, Kim et al. [40] observed non-monotonic dose-dependent alterations in DNA methylation among mouse liver samples from offspring exposed perinatally to multiple doses of BPA through the maternal diet. These changes may provide insight regarding a mechanism of action for BPA during developmental exposure; however, further information regarding phenotypic changes is necessary to determine whether epigenetic changes at low level exposures are significant indicators of a dose-disease response relationship. Thus, *biological gradient* can be broadened to include molecular dose–response relationships, if the actual response occurs at a dose that is also associated with disease onset or progression.

## Criteria 6: plausibility

Even at the time it was introduced, *biological plausibility* represented fundamental concepts of data integration—the criterion implies that epidemiology and biology must interact [5]. Plausibility has historically been judged based on the presence of existing biological or social models that explain the association of interest. Hill’s criterion of plausibility is satisfied if the relationship is consistent with the current body of knowledge regarding the etiology and mechanism of disease; though, Hill admitted that this interpretation of *biological plausibility* was dependent on the current state of knowledge. Today, tools such as high-throughput screening assays can be used to study a specific biologically plausible pathway and identify toxic agents that interfere with that pathway in defined ways. Indeed, opening the ‘black box’ through integrating molecular epidemiological advancements has allowed researchers to illuminate more steps in the exposure-to-effect paradigm, contributing to an understanding of biological plausibility for suggested causal relationships.

The elucidation of biological pathways leading to liver toxicity have played a large role in advancing the interpretation of *biological plausibility*, and the integration of knowledge from various evidence streams has aided in those interpretations. The liver is typically the first organ with appreciable capacity for oxidative metabolism that an agent encounters after ingestion, and is therefore a key organ for studying potential toxicity [16]. Liver effects demonstrated using techniques such as high-throughput in vitro and in silico cell manipulation, can be seen as a harbinger for further toxic endpoints that might occur with more refined, realistic exposures [41, 42]. However, as demonstrated by the newly-developed “virtual liver” [43], the future of testing biological plausibility likely lies with in silico experimentation. Researchers can now predict plausible relationships using in vitro and in silico screening tools targeting defined disease mechanisms, which represents a potential paradigm shift in how scientists frame causal research questions and design studies.

Historically, causal inference was approached with the assumption of a single-factor direct relationship (i.e. A causes B). However, researchers now understand that many disease outcomes are a result of the interplay and balance between multiple contributing and intermediary factors. As such, demonstrating the biological plausibility of a causal relationship can be complex. However, improved statistical techniques can help researchers to understand complex disease progression from a molecular standpoint, where multiple risk factors, confounders, adaptive responses, and mediating mechanisms intersect [44–46]. For example, the biostatistical approach of mediation analysis allows for the disentanglement and decomposition of the various biological pathways of direct and indirect effects that play a role in filling the “black box” between exposures and observable outcomes [47].

## Criteria 7: coherence

*Coherence* has been viewed as being similar to *biological plausibility*, in that the cause-and-effect story should make sense with all knowledge available to the researcher, and this criterion has not changed greatly since its inception. Indeed, Hill identified histopathological evidence of bronchial epithelium changes and animal-based toxicity tests for the carcinogenicity of cigarette smoke as an example of a coherent story among several avenues of study design. Today, *coherence* is another area in which molecular-based studies have been used to demonstrate a comprehensible story regarding various aspects of the exposure-to-disease paradigm. For example, lung tissue fiber analysis by scanning transmission electron microscopy (STEM) has expanded our knowledge of internal biologically effective amphibole dose relating to altered structure and function of lung tissue, supporting the conclusion that amphibole asbestos fibers induce mesothelioma [48].

Alternatively, advanced mechanistic studies can elucidate an incoherent body of epidemiologic literature, thereby strengthening the causal inference in one direction or another. Consider for example the carcinogenicity of hexavalent chromium [Cr(VI)]. The body of epidemiologic literature regarding the carcinogenicity of Cr(VI) is limited and conflicting, particularly regarding ingestion exposures (e.g., drinking water) and cancers outside the respiratory system (e.g., cancers of the GI tract). However, a recent array of genomic, pharmacokinetic, and mechanistic research—including metabolism, bioavailability and kinetic studies, mutagenic mode of action studies, and gene expression profiling—demonstrate that ingested Cr(VI) does indeed have a carcinogenic profile [49, 50].

## Criteria 8: experiment

Hill explained that evidence drawn from experimental manipulation—particularly epidemiologic studies in disease risk declines following an intervention or cessation of exposure—may lead to the strongest support for causal inference. Yet in modern contexts, experimentation must consider that many diseases result from multifaceted exposures and follow complex progression pathways. Cessation of exposure as Hill described may not reverse or appreciably slow the progression of disease. In some cases, multiple risk factors, including diet, exercise, smoking, chemical exposures, and genetic predisposition can contribute to disease onset and progression. Thus, while the combination of these factors may culminate in disease, experimental manipulation of a single contributory factor may or may not result in observable decreases in disease incidence.

Researchers using a data integration framework can now draw from toxicological findings for experimental insight into causality. In vitro studies that test mechanistic pathways and demonstrate the biological role of an agent in disease progression may result in knowledge that can be used to predict potential human health outcomes in a much more time-efficient manner than human studies, particularly for adverse outcomes with a long latency period.

The expanded understanding of *temporality* in light of data from varied evidence streams can also affect interpretation of the *experiment* criterion. Individual exposures can cause epigenetic modifications to parental DNA that result in an observed effect in future offspring, even though there is no direct exposure to the offspring. Experimental studies in animal models are often necessary to provide mechanistic support for an epidemiologic observation that involves complex temporality. For example, multiple animal studies provide support for the hypothesis that epigenetic changes induced by DES exposure in utero may be causative of transgenerational effects of DES exposure in females [32, 51–54]. Because epigenetic analyses in transgenerational human studies take decades and are riddled with potential confounders, reliance on animal models and advanced analytical techniques can help to support determination of a causal relationship.

## Criteria 9: analogy

Hill implied that when there is strong evidence of a causal relationship between a particular agent and a specific disease, researchers should be more accepting of weaker evidence that a similar agent may cause a similar disease. *Analogy* has been interpreted to mean that when one causal agent is known, the standards of evidence are lowered for a second causal agent that is similar in some way [55]. Some modern epidemiologists have argued that a lack of analogy does not preclude causation, but simply implies a lack of creativity on the researcher’s part [56]. Indeed, some might argue that enough knowledge exists and is accessible today to identify an analogy for every situation, especially if the researcher pulls that knowledge from multiple disciplines and across evidence streams. Today, researchers have a wider range of tools by which to seek an analogy, including disease progression pattern, common risk factors and confounders, and biological mechanisms of action. Therefore, the modern value of *analogy* is not gained from confirming a causal inference, but rather from proposing and testing mechanistic hypotheses.

As an example, analogous mechanistic hypothesis testing has been conducted on carbon nanotubes (CNTs) using the extensive literature on the mechanistic toxicity of asbestos fibers. Models based on molecular structure and physical–chemical characteristics such as aspect ratio predict a mechanism of action similar to that of asbestos [57]. The physical morphology of CNTs appears similar to that of asbestos fibers; thus, respirable-sized fibers are expected to behave similarly in occupational settings and lead to similar lung translocation and deposition. Additionally, asbestos fibers are known to cause inflammation and fibrosis of the lung pleura as a precursor to mesothelioma; these same outcomes have been demonstrated following CNT exposure [58, 59]. Further, CNTs have been found to stimulate the release of acute phase cytokines from human macrophages and mesothelial cells exposed to CNTs of varying lengths, demonstrating that CNT exposure results in a length-dependent pro-inflammatory response, similar to that of asbestos [60]. These findings enhance the asbestos analogy by confirming that CNTs may be capable of causing disease that begins with pleural inflammation—the same mechanism responsible for asbestos-related mesothelioma. However, the results also demonstrate that not all CNTs have the same potential for carcinogenicity, implying that proactive design of engineered CNTs can limit the risks and allow for safe use of the compounds in a variety of applications—and that the analogy to asbestos should not be viewed in a way that limits continued research.

## Conclusion

Hill’s nine aspects of association were never intended to be viewed as rigid criteria or as a checklist for causation, yet have been popularized as such over the past 50 years. Instead, the so called “Bradford Hill Criteria” were written as flexible guidelines or considerations meant to guide epidemiologic investigations and aid in causal inference. As the world of epidemiologic research has changed and expanded, our criteria for determining causal inference must similarly evolve. As Chen and Hunter explained, researchers today are “much more of a participant in the assessment of the biologic basis for an association, by using biologic measurements to assess exposure, internal dose, biologically effective dose, early biologic effect, altered structure/function, invasive cancer diagnosis, tumor metastasis and prognosis”—essentially, the ‘black box’ between exposure and disease can now be peered into and explored [2]. Epidemiologic investigation of causation conducted today must also evolve to reflect the concepts of data integration. This involves incorporating not just traditional epidemiological evidence but also evidence gathered by opening the ‘black box’ and incorporating data from molecular biology, toxicology, genotoxicology, and other disciplines into evaluations of causation. The advanced tools and techniques that have developed in recent decades across all scientific disciplines have affected the application and interpretation of the Bradford Hill criteria, which were originally written to fit the ‘black box’ model of epidemiologic studies.

The Bradford Hill Criteria remain one of the most cited concepts in health research and are still upheld as valid tools for aiding causal inference [61]. However, the way each criterion should be applied, interpreted, and weighted in a data integration framework must be carefully measured against the varied and often novel types of data available in each unique situation. In some ways, data integration degrades the value and importance of certain criteria, as it offers alternative interpretations for each criterion that give way for inductivism. In other words, in a data integration framework, researchers can interpret the criterion whichever way fits the available data as opposed to determining whether the data meets the criterion. This type of application is dangerous as it bypasses the ultimate purpose of causal inference—determining whether the observed association is directionally causal or not.

Nonetheless, data integration represents an opportunity to expand our abilities as researchers to think about causation. Herein, we have discussed how the data integration framework requires the compilation of more lines of evidence and more scrutiny for each of the criteria. The examples above have demonstrated that data integration can enhance the application of the Bradford Hill Criteria in a causal analysis by: allowing for more scrutiny in study designs; providing new tools to demonstrate consistency, specificity, and plausibility of associations; integrating molecular evaluation to determine temporality and dose–response; clarifying conflicting epidemiologic findings to determine coherence; and promoting the proposal and testing of new mechanistic hypotheses.

The Bradford Hill Criteria are far from outdated in a data integration framework. Causal inference in the field of epidemiology is no longer informed solely by traditional epidemiologic studies, but rather by a complementary host of evolving research tools and scientific disciplines. Although specific interpretations of each criterion have evolved over time, the concepts that underlie each criterion can be applied to a variety of methodologies to answer questions about causation. The Bradford Hill Criteria can aid researchers in connecting the dots within a body of literature, either to lead to suggestions of causal relationships or identification of what more research is needed to understand potential causality. As ever, the criteria should not be used as a heuristic for assessing causation in a vacuum; rather they should be viewed as a list of possible considerations meant to generate thoughtful discourse among researchers from diverse scientific fields. The interpretive concepts we have introduce into each Bradford Hill criterion in light of data integration support the Bradford Hill Criteria’s function as a valid and useful tool when establishing causation.

## References

[1] Hill AB (1965). The environment and disease: association or causation?. Proc R Soc Med..

[2] Chen YC, Hunter DJ (2005). Molecular epidemiology of cancer. CA Cancer J Clin.

[3] Geneletti S, Gallo V, Porta M, Khoury MJ, Vineis P (2011). Assessing causal relationships in genomics: from Bradford-Hill criteria to complex gene-environment interactions and directed acyclic graphs. Emerg Themes Epidemiol.

[4] Porta M (2008). A dictionary of epidemiology.

[5] Re Porta M (1999). Biologic plausibility in causal inference: current method and practice. Am J Epidemiol.

[6] Real FX, Bertranpetit J, Estrivill X (2000). Genes as causes: scientific fact or simplistic thinking?. J Epidemiol Commun Health.

[7] Porta M, Alvarez-Dardet C (2000). Authors’ reply: how is causal inference practised in the biological sciences?. J Epidemiol Commun Health.

[8] Rooney AA, Boyles AL, Wolfe MS, Bucher JR, Thayer KA (2014). Systematic review and evidence integration for literature-based environmental health science assessments. Environ Health Perspect.

[9] Doerrer NG (2007). Integration of human biomonitoring exposure data into risk assessment: HESI initiatives and perspectives. Int J Hyg Environ Health.

[10] Adami H-O, Berry SCL, Breckenridge CB, Smith LL, Swenberg JA, Trichopoulos D (2011). Toxicology and epidemiology: improving the science with a framework for combining toxicological and epidemiological evidence to establish causal inference. Toxicol Sci.

[11] ECETOC. Technical Report No. 104: Framework for the integration of human and animal data in chemical risk assessment. European Centre for Ecotoxicology and Toxicology of Chemicals; 2009.

[12] Ronk CJ, Hollins DM, Jacobsen MJ, Galbraith DA, Paustenbach DJ (2013). Evaluation of pulmonary function within a cohort of flavorings workers. Inhal Toxicol.

[13] NIOSH. Lung function (Spirometry) testing in employees at a flavorings manufacturing plant—Indiana. Centers for Disease Control and Prevention; National Institute for Occupational Safety and Health. Atlanta, GA 2011.

[14] Fitzmamice G, Laird NM, Ware JH (2004). Applied longitudinal analysis.

[15] Ware JH (1985). Linear models for the analysis of longitudinal studies. Am Stat.

[16] Klaassen CD, Casarett LJ (2007). Casarett and Doull’s toxicology: the basic science of poisons.

[17] Gross SA, Zheng JH, Le AT, Kerzic PJ, Irons RD (2006). PU.1 phosphorylation correlates with hydroquinone-induced alterations in myeloid differentiation and cytokine-dependent clonogenic response in human CD34(+) hematopoietic progenitor cells. Cell Biol Toxicol.

[18] Lauby-Secretan B, Loomis D, Grosse Y, El Ghissassi F, Bouvard V, Benbrahim-Tallaa L (2013). Carcinogenicity of polychlorinated biphenyls and polybrominated biphenyls. Lancet Oncol.

[19] Luecke S, Backlund M, Jux B, Esser C, Krutmann J, Rannug A (2010). The aryl hydrocarbon receptor (AHR), a novel regulator of human melanogenesis. Pigment Cell Melanoma Res.

[20] IARC. IARC monographs on the evaluation of carcinogenic risks to humans. Polychlorinated and polybrominated biphenyls, vol 107. Lyon: International Agency for Research on Cancer (in press).

[21] Roggli VL, Gibbs AR, Attanoos R, Churg A, Popper H, Cagle P (2010). Pathology of asbestosis- an update of the diagnostic criteria: report of the asbestosis committee of the college of american pathologists and pulmonary pathology society. Arch Pathol Lab Med.

[22] Hesterberg TW, Chase G, Axten C, Miller WC, Musselman RP, Kamstrup O (1998). Biopersistence of synthetic vitreous fibers and amosite asbestos in the rat lung following inhalation. Toxicol Appl Pharmacol.

[23] Churg A, Wright JL, Vedal S (1993). Fiber burden and patterns of asbestos-related disease in chrysotile miners and millers. Am Rev Respir Dis.

[24] Churg A, Vedal S (1994). Fiber burden and patterns of asbestos-related disease in workers with heavy mixed amosite and chrysotile exposure. Am J Respir Crit Care Med.

[25] Rothman KJ, Greenland S (2005). Causation and causal inference in epidemiology. Am J Public Health.

[26] Gruber JF, Karagas MR, Gilbert-Diamond D, Bagley PJ, Zens MS, Sayarath V (2012). Associations between toenail arsenic concentration and dietary factors in a New Hampshire population. Nutr J.

[27] Wright RO, Amarasiriwardena C, Woolf AD, Jim R, Bellinger DC (2006). Neuropsychological correlates of hair arsenic, manganese, and cadmium levels in school-age children residing near a hazardous waste site. Neurotoxicology.

[28] Cleland B, Tsuchiya A, Kalman DA, Dills R, Burbacher TM, White JW (2009). Arsenic exposure within the Korean community (United States) based on dietary behavior and arsenic levels in hair, urine, air, and water. Environ Health Perspect.

[29] Steinmaus C, Ferreccio C, Acevedo J, Yuan Y, Liaw J, Duran V (2014). Increased lung and bladder cancer incidence in adults after in utero and early-life arsenic exposure. Cancer Epidemiol Biomarkers Prev.

[30] Melak D, Ferreccio C, Kalman D, Parra R, Acevedo J, Perez L (2014). Arsenic methylation and lung and bladder cancer in a case-control study in northern Chile. Toxicol Appl Pharmacol.

[31] Dolinoy DC, Weidman JR, Jirtle RL (2007). Epigenetic gene regulation: linking early developmental environment to adult disease. Reprod Toxicol.

[32] Hilakivi-Clarke L (2014). Maternal exposure to diethylstilbestrol during pregnancy and increased breast cancer risk in daughters. Breast Cancer Res.

[33] Hoyo C, Murphy SK, Jirtle RL (2009). Imprint regulatory elements as epigenetic biosensors of exposure in epidemiological studies. J Epidemiol Community Health.

[34] Skaar DA, Li Y, Bernal AJ, Hoyo C, Murphy SK, Jirtle RL (2012). The human imprintome: regulatory mechanisms, methods of ascertainment, and roles in disease susceptibility. ILAR J.

[35] Swaen G, van Amelsvoort L (2009). A weight of evidence approach to causal inference. J Clin Epidemiol.

[36] Calabrese EJ, Blain RB (2011). The hormesis database: the occurrence of hormetic dose responses in the toxicological literature. Regul Toxicol Pharmacol.

[37] Calabrese EJ (2013). Hormetic mechanisms. Crit Rev Toxicol.

[38] Szabadi E (1977). A model of two functionally antagonistic receptor populations activated by the same agonist. J Theor Biol.

[39] Barlow CA, Lievense L, Gross S, Ronk CJ, Paustenbach DJ (2013). The role of genotoxicity in asbestos-induced mesothelioma: an explanation for the differences in carcinogenic potential among fiber types. Inhal Toxicol.

[40] Kim JH, Sartor MA, Rozek LS, Faulk C, Anderson OS, Jones TR (2014). Perinatal bisphenol A exposure promotes dose-dependent alterations of the mouse methylome. BMC Genom.

[41] Alfirevic A, Gonzalez-Galarza F, Bell C, Martinsson K, Platt V, Bretland G (2012). In silico analysis of HLA associations with drug-induced liver injury: use of a HLA-genotyped DNA archive from healthy volunteers. Genome Med.

[42] Kavlock R, Dix D (2010). Computational toxicology as implemented by the US EPA: providing high throughput decision support tools for screening and assessing chemical exposure, hazard and risk. J Toxicol Environ Health B Crit Rev.

[43] U.S. EPA. The Virtual Liver Project. 2014. http://www.epa.gov/ncct/virtual_liver/. Accessed 24 Jun 2014.

[44] Albert JM, Nelson S (2011). Generalized causal mediation analysis. Biometrics.

[45] Pearl J (2010). On the consistency rule in causal inference: axiom, definition, assumption, or theorem?. Epidemiology.

[46] Vanderweele TJ (2012). Mediation analysis with multiple versions of the mediator. Epidemiology.

[47] Richiardi L, Bellocco R, Zugna D (2013). Mediation analysis in epidemiology: methods, interpretation and bias. Int J Epidemiol.

[48] Rodelsperger K, Woitowitz HJ, Bruckel B, Arhelger R, Pohlabeln H, Jockel KH (1999). Dose-response relationship between amphibole fiber lung burden and mesothelioma. Cancer Detect Prev.

[49] Zhitkovich A (2011). Chromium in drinking water: sources, metabolism, and cancer risks. Chem Res Toxicol.

[50] Antonova L, Aronson K, Mueller CR (2011). Stress and breast cancer: from epidemiology to molecular biology. Breast Cancer Res.

[51] Skinner MK, Manikkam M, Tracey R, Guerrero-Bosagna C, Haque M, Nilsson EE (2013). Ancestral dichlorodiphenyltrichloroethane (DDT) exposure promotes epigenetic transgenerational inheritance of obesity. BMC Med.

[52] Doherty LF, Bromer JG, Zhou Y, Aldad TS, Taylor HS (2010). In utero exposure to diethylstilbestrol (DES) or bisphenol-A (BPA) increases EZH2 expression in the mammary gland: an epigenetic mechanism linking endocrine disruptors to breast cancer. Horm Cancer.

[53] Jefferson WN, Chevalier DM, Phelps JY, Cantor AM, Padilla-Banks E, Newbold RR (2013). Persistently altered epigenetic marks in the mouse uterus after neonatal estrogen exposure. Mol Endocrinol.

[54] Kaati G, Bygren LO, Edvinsson S (2002). Cardiovascular and diabetes mortality determined by nutrition during parents’ and grandparents’ slow growth period. Eur J Hum Genet.

[55] Susser M (1991). What is a cause and how do we know one? A grammar for pragmatic epidemiology. Am J Epidemiol.

[56] Hofler M (2005). The Bradford Hill considerations on causality: a counterfactual perspective. Emerg Themes Epidemiol.

[57] Donaldson K, Murphy FA, Duffin R, Poland CA (2010). Asbestos, carbon nanotubes and the pleural mesothelium: a review of the hypothesis regarding the role of long fibre retention in the parietal pleura, inflammation and mesothelioma. Part Fibre Toxicol.

[58] Schinwald A, Murphy FA, Prina-Mello A, Poland CA, Byrne F, Movia D (2012). The threshold length for fiber-induced acute pleural inflammation: shedding light on the early events in asbestos-induced mesothelioma. Toxicol Sci.

[59] Murphy FA, Poland CA, Duffin R, Al-Jamal KT, Ali-Boucetta H, Nunes A (2011). Length-dependent retention of carbon nanotubes in the pleural space of mice initiates sustained inflammation and progressive fibrosis on the parietal pleura. Am J Pathol.

[60] Murphy FA, Schinwald A, Poland CA, Donaldson K (2012). The mechanism of pleural inflammation by long carbon nanotubes: interaction of long fibres with macrophages stimulates them to amplify pro-inflammatory responses in mesothelial cells. Part Fibre Toxicol.

[61] Phillips CV, Goodman KJ (2004). The missed lessons of Sir Austin Bradford Hill. Epidemiol Perspect Innov.

